# Supplementary material for: A methodological approach for inferring causal relationships from opinions and news-derived events with an application to climate change
Source: PeerJ Comput Sci. 2025 Jun 19;11:e2964. doi: 10.7717/peerj-cs.2964 (PMC12193450; doi:10.7717/peerj-cs.2964)
Supplement: Supplemental Information 4 — An overview of frequent itemset and association rule mining. Application to climate change data analysis. [file peerj-cs-11-2964-s004.pdf]

Supplementary material for: A methodological approach for inferring causal relationships from opinions and news-derived events with an application to climate change

Frequent Itemset and Association Rules Mining

Frequent itemset and association rule mining aims to identify relationships among various variables, indicating which sets of tags frequently appear together in a tweet. Additionally, it seeks to determine the conditions under which the presence of a specific tag or set of tags increases the likelihood of another tag being present. In this analysis, the tweets are examined individually, without grouping them by time period. Given an itemset C, its support is defined as the number of individual tweets that contain C. An itemset C is considered frequent if its support exceeds a specified minimum support threshold a.

Association rules establish a relationship between an itemset C and an item i. This relationship, denoted as $C \rightarrow i$, implies that if the itemset C is present in a tweet, it is significantly likely that i will also be present. The confidence of a rule $C \rightarrow i$ is defined as the ratio of the support of $C \cup \{i\}$ to the support of C. For example, if the rule {X, Y} $\rightarrow$ Z has a confidence of 0.35, this indicates that among tweets labeled as 'X' and 'Y', 35% are also labeled as 'Z'. The A-Priori [Agrawal, 1994] algorithm addresses the problem of finding frequent itemsets by making efficient use of system memory. It intelligently stores the occurrence counts of each itemset, avoiding memory allocation for itemsets that cannot be frequent.

The following metrics are used to assess the strength and relevance of an association rule of the form $C \rightarrow i$, where C is a set of items and i is a single item associated with a dataset of tweets:

- **Support** indicates the proportion of tweets in the dataset that contain both the itemset C and the item i. It is calculated as:

$$\text{Support}(C \rightarrow i) = \frac{\text{Number of tweets containing } C \cup \{i\}}{\text{Total number of tweets}}$$

  Support measures the frequency of occurrence for the rule $C \rightarrow i$ within the dataset, highlighting common itemsets.

- **Confidence** measures the likelihood that the item i appears in tweets that already contain C. For the rule $C \rightarrow i$, it is calculated as:

$$\text{Confidence}(C \rightarrow i) = \frac{\text{Support}(C \rightarrow i)}{\text{Support}(C)}$$

  Confidence evaluates the predictive power of the rule, or how likely it is to find i in tweets that contain C.

- **Lift** provides insight into the strength of the association by comparing the observed support of $C \rightarrow i$ to the support expected if C and i were independent. For the rule $C \rightarrow i$, lift is defined as:

$$\text{Lift}(C \to i) = \frac{\text{Support}(C \to i)}{\text{Support}(C) \times \text{Support}(i)}$$

A lift greater than 1 suggests a positive association between C and i; a lift less than 1 suggests a negative association, and a lift of 1 implies independence. For example, a lift of 1.5 implies that, given C, the probability of i occurring is 50% higher than if C and i were independent.

Frequent Itemsets and Association Rules in Climate Change Data Analysis

An analysis of frequent itemsets and association rules was conducted on the tweet dataset. In this study, we used an implementation of the A-Priori algorithm from the Python library efficient-apriori [Odland, 2024], which computes both frequent itemsets and association rules. For the algorithm's execution, a minimum support threshold of 1% and a minimum confidence threshold of 35% were set. These parameters reduce the number of itemsets and rules produced, excluding those with low support or confidence, which are considered less meaningful.

The output from running the A-Priori algorithm on the tweet dataset is a list of frequent itemsets. For each itemset, details are provided, including the itemset itself (in this case represented as a tuple of tags), its size, and its support, which indicates the percentage of tweets that are simultaneously tagged with all tags in the itemset. The size-1 itemsets are presented in Table 1. Although these itemsets are not particularly useful for analyzing relationships among different tags, they do indicate the proportion of tweets covered by each tag individually.

**Itemset**	**Support**
Non-Aggressive	0.7106
Believer	0.7071
Neutral Sentiment	0.4118
Negative Sentiment	0.3119
[T] Global Stance	0.2855
Positive Sentiment	0.2763
Neutral	0.2131
[T] Importance of Human Intervention	0.1792
[T] Weather Extremes	0.1702
[T] Politics	0.1249
Denier	0.0797
[T] Denialist Politicians versus Science	0.0688
[T] Severity of Gas Emissions	0.0624
[T] Ideological Positions on Global Warming	0.0416
[T] Impact of Resource Overconsumption	0.0343
[T] Significance of Pollution Awareness Events	0.0331

Table 1: Frequent itemsets of size 1

A total of 17 frequent itemsets of size 1 are identified, corresponding to the number of original tags, as each tag appears in at least 1% of the tweets, thus exceeding the

minimum support threshold set for the algorithm's execution. The tags 'Non-Aggressive' and 'Believer' are the two most common, each present in over 70% of tweets. The distribution of topics is also worth noting, with 'Global Stance' as the most frequently occurring topic. Finally, we observe that the percentage of tweets labeled as 'Denier' is relatively low, at just under 8%. Table 2 displays the frequent itemsets of size 2 with the highest support. These sets could be useful for an initial analysis of relationships among the various tags, as they include more than one.

**Itemset**	**Support**
Believer, Non-Aggressive	0.5105
Neutral Sentiment, Non-Aggressive	0.293
Believer, Neutral Sentiment	0.288
Believer, [T] Global Stance	0.236
Non-Aggressive, [T] Global Stance	0.2213
Believer, Negative Sentiment	0.2143
Believer, Positive Sentiment	0.2048
Aggressive, Believer	0.1966
Negative Sentiment, Non-Aggressive	0.1963
Neutral, Non-Aggressive	0.1539
Believer, [T] Importance of Human Intervention	0.1427
Non-Aggressive, [T] Importance of Human Intervention	0.1288
Non-Aggressive, [T] Weather Extremes	0.1277
Aggressive, Neutral Sentiment	0.1188
Neutral Sentiment, [T] Global Stance	0.118
...	...

Table 2: Frequent itemsets of size 2

The A-Priori algorithm identified 79 itemsets of size 2 that exceed the 1% support threshold (Table 2 presents only the top 15). By examining the initial sets in the table, it is evident that the labels 'Non-Aggressive' and 'Believer' are present in all 14 sets with the highest support. This outcome is expected, as both labels individually appear in over 70% of the tweets. However, they do not provide particularly relevant information. For instance, the set {'Believer', '[T] Global Stance'} has a support of 23%, yet it is challenging to determine from this analysis alone whether there is a genuine relationship between these labels or if this result is simply due to the 'Believer' label appearing in more than 70% of the tweets. This high frequency could make the combination of this label with other frequent labels relatively common.

Similar analyses were conducted with itemsets of larger sizes. Some of the itemsets of size 3 with highest support are {'Aggressive', 'Negative Sentiment', '[T] Global Stance'}, with a support of 2.18% and {'Aggressive', 'Negative Sentiment, '[T] Politics'}, with a support of 2.16%. While the results allow for some insights into how different labels may relate, drawing conclusions remains challenging since high support alone does not necessarily indicate a strong relationship between the labels in a set. For this reason, frequent itemset analysis alone may not be ideal for examining relationships among the different labels. However, it serves as a necessary step for discovering association rules, which are discussed next.

The association rules identified were divided into three groups of interest based on the various values for the measures previously described. These groups of rules are presented

below using a graph representation, where the source nodes represent antecedents, the target nodes represent consequents, and the weights associated with the edges represent the confidence of the rules.

The first group of rules, presented in the graph of Figure 1, includes all rules that meet the following conditions:

- Support > 0.02: the antecedent and consequent are present simultaneously within the dataset in at least 2% of cases.
- Confidence > 0.4: the rule holds true in at least 40% of cases.
- Lift > 1.45: given the antecedent, the consequent is at least 45% more likely than if they were independent.

This group includes relatively frequent rules with a lift that makes them quite significant. Notable rules in this group include:

- {'Positive Sentiment', '[T] Weather Extremes'} → 'Neutral': this rule has a confidence of 55%, meaning that of the tweets labeled as 'Positive Sentiment' and '[T] Weather Extremes,' 55% are also labeled as 'Neutral'. This is particularly interesting given that only 20% of the total tweets are labeled as 'Neutral'.
- {'Deniers'} → 'Aggressive' and {'Denier'} → 'Negative Sentiment': these findings align with the exploratory analysis, where deniers were observed to be more aggressive and to express a more negative sentiment.
- {'Believer', 'Non-Aggressive', '[T] Global Stance'} → 'Positive Sentiment': this is the only rule in this group with 'Positive Sentiment' as the consequent, whereas the majority of rules found have 'Negative Sentiment' or 'Aggressiveness' as the consequent.

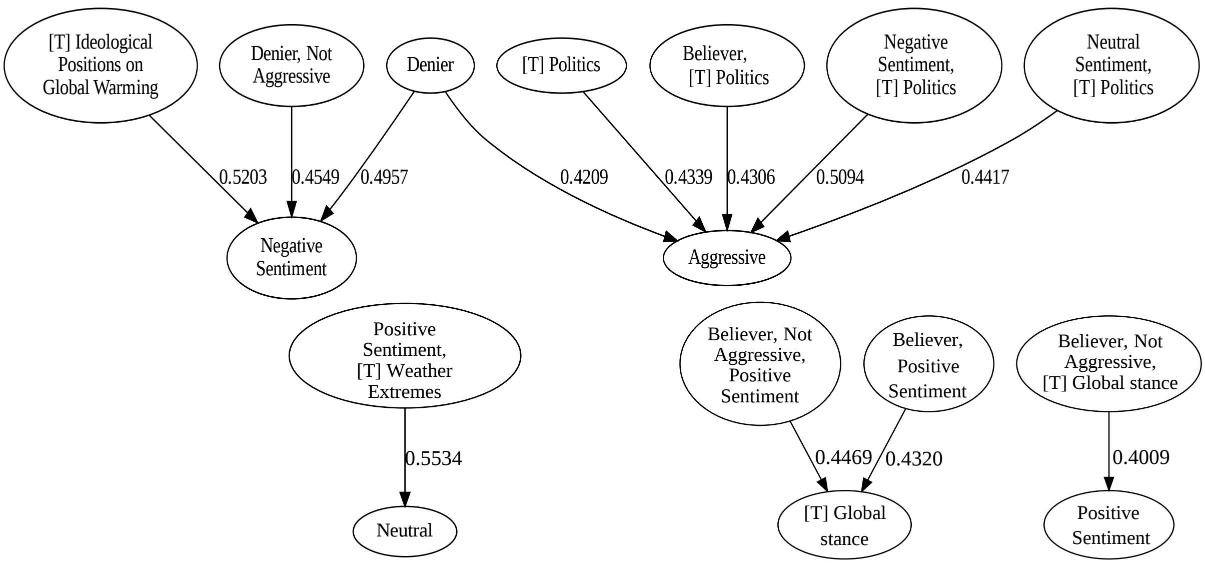

Figure 1: Graph representing association rules with Support > 0.02, Confidence > 0.4, and Lift > 1.45.

For the second group of rules, presented in the graph of Figure 2, the minimum support was reduced to 0.01, and the minimum confidence was increased to 0.5. This adjustment

results in a set of rules that may not be as frequent but describe associations where, given the antecedent, there is more than a 50% probability that the consequent also holds. Particularly noteworthy is the rule {'Believer', 'Non-Aggressive', '[T] Ideological Positions on Global Warm → 'Negative Sentiment', which has a confidence of 0.68, making it the rule with the highest confidence among the three groups, occurring in 68% of the cases.

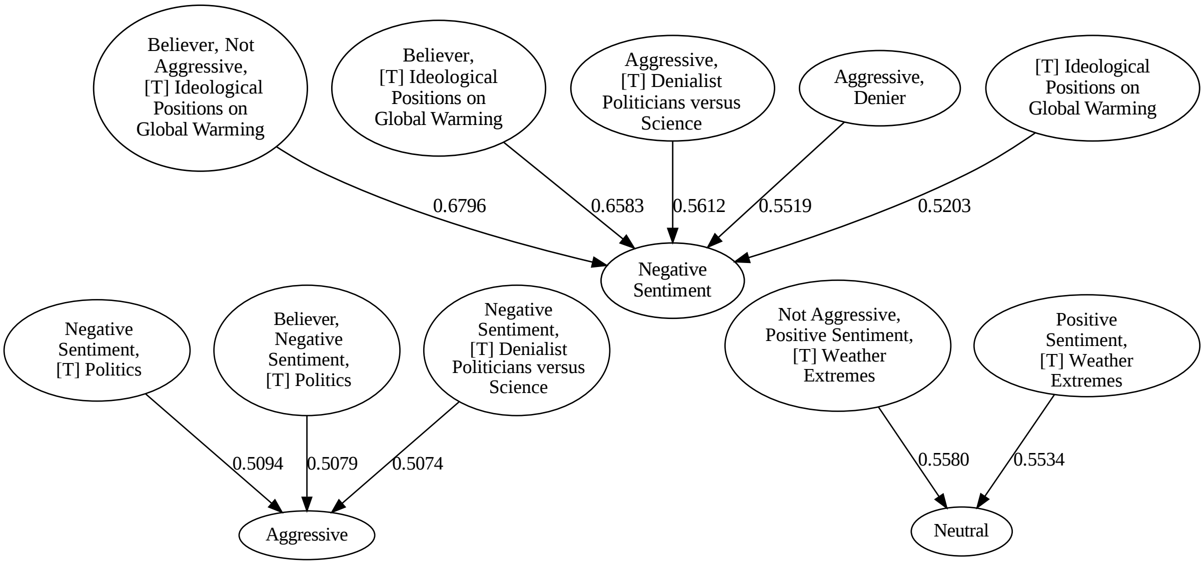

Figure 2: Graph representing association rules with Support > 0.01, Confidence > 0.5, and Lift > 1.45.

Finally, for the third group of rules, presented in the graph of Figure 3, a minimum support of 0.02, a minimum confidence of 0.35, a minimum lift of 1.25, and the restriction that the antecedent consists of only one label were established. This results in a set of rules that may generally not be as strong but are easier to analyze, as they illustrate relationships between pairs of labels. From this group, the bidirectional relationship between 'Positive Sentiment' and '[T] Global Stance' is particularly noteworthy, as well as the rules {'[T] Denialist Politicians versus Science'} → 'Negative Sentiment' and '[T] Denialist Politicians versus Science'} → 'Aggressiveness', which demonstrate a similar behavior between the topic Denialist Politicians versus Science and the denier stance concerning negative sentiment and aggressiveness. It is worth noting that the topic Denialist Politicians versus Science is the topic with the highest number of denialist tweets.

References

R Agrawal. Fast algorithms for mining association rules. VLDB, 1994.

Tommy Odland. Efficient-Apriori: An efficient implementation of the apriori algorithm, 2024. URL https://github.com/tommyod/Efficient-Apriori. Accessed: 2024-11-11.

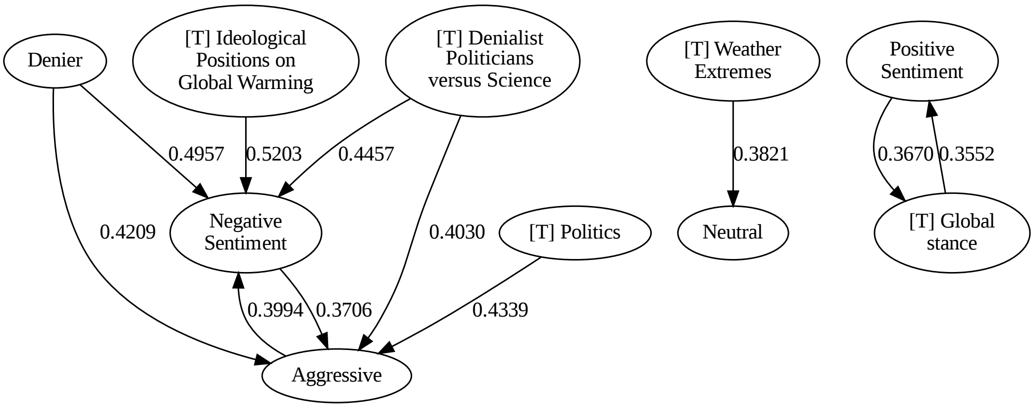

Figure 3: Graph representing association rules with Support > 0.02, Confidence > 0.35, Lift > 1.25, and Maximum Size = 2.
